# Hypoxia treatment and resistance training alters microRNA profiling in rats skeletal muscle

**DOI:** 10.1038/s41598-024-58996-7

**Published:** 2024-04-10

**Authors:** Tao Mei, Yang Hu, Ying Zhang, Yanchun Li

**Affiliations:** China Institute of Sport and Health Science, Beijing Sport University, Beijing, China

**Keywords:** Hypoxia, Resistance training, Skeletal muscle atrophy, MiRNA expression profile, Bioinformatics analysis, RNA, Gene regulatory networks, Musculoskeletal system, Gene regulation, Non-coding RNAs

## Abstract

MicroRNAs (miRNAs) may play a crucial regulatory role in the process of muscle atrophy induced by high-altitude hypoxia and its amelioration through resistance training. However, research in this aspect is still lacking. Therefore, this study aimed to employ miRNA microarray analysis to investigate the expression profile of miRNAs in skeletal muscle from an animal model of hypoxia-induced muscle atrophy and resistance training aimed at mitigating muscle atrophy. The study utilized a simulated hypoxic environment (oxygen concentration at 11.2%) to induce muscle atrophy and established a rat model of resistance training using ladder climbing, with a total intervention period of 4 weeks. The miRNA expression profile revealed 9 differentially expressed miRNAs influenced by hypoxia (e.g., miR-341, miR-32-5p, miR-465-5p) and 14 differentially expressed miRNAs influenced by resistance training under hypoxic conditions (e.g., miR-338-5p, miR-203a-3p, miR-92b-3p) (∣log2(FC)∣ ≥ 1.5, *p* < 0.05). The differentially expressed miRNAs were found to target genes involved in muscle protein synthesis and degradation (such as Utrn, mdm2, eIF4E), biological processes (such as negative regulation of transcription from RNA polymerase II promoter, regulation of transcription, DNA-dependent), and signaling pathways (such as Wnt signaling pathway, MAPK signaling pathway, ubiquitin-mediated proteolysis, mTOR signaling pathway). This study provides a foundation for understanding and further exploring the molecular mechanisms underlying hypoxia-induced rats muscle atrophy and the mitigation of atrophy through resistance training.

## Introduction

During the period of acclimatization when populations residing in lowland areas transition to high-altitude regions, the distinctive environmental stimuli of the elevated terrain may result in a certain degree of skeletal muscle atrophy^1^, potentially leading to adverse effects on muscle functionality. Hypoxia is acknowledged as one of the contributing factors to skeletal muscle atrophy in high-altitude environments, alongside factors such as reduced atmospheric pressure and dietary shifts. Engaging in resistance training has been identified as beneficial for mitigating muscle atrophy. In animal models, resistance training has shown the capacity to alleviate skeletal muscle atrophy induced by various factors, including hindlimb immobilization^2^, dexamethasone^3^, diabetes^4^, aging^5^, and chronic obstructive pulmonary disease. The mechanisms by which both hypoxia and resistance training influence skeletal muscle mass primarily entail disturbances in the balance between protein synthesis and degradation. For example, under hypoxic conditions, disruptions in signaling pathways such as Akt/mTOR and the ubiquitin‒proteasome system (UPS) within skeletal muscle have been observed^6,7^. It is plausible that other signaling pathways also play a role in regulating skeletal muscle atrophy; however, further elucidation is needed regarding the regulation of gene expression within these pathways.

MicroRNAs (miRNAs) are a class of noncoding RNAs that are distinct from long noncoding RNAs (lncRNAs). With remarkably short chain lengths, typically spanning approximately 22 nucleotides, miRNAs exert a significant influence on gene regulation despite their brevity. Through complementary base pairing with target gene nucleotide sequences, miRNAs orchestrate the modulation of gene expression. Within the context of skeletal muscle growth and development, miRNAs assume critical roles in regulating diverse processes encompassing muscle cell proliferation, differentiation, hypertrophy, atrophy, and transitions in muscle fiber types^8,9^.

In the realm of muscle atrophy regulation, research has unveiled a spectrum of miRNAs implicated in this intricate process. Notable examples include miR-455-3p, let-7d-3p, and miR-376c-3p, among others, which exert regulatory control over muscle quality in the context of aging^10–12^. Similarly, miR-424-5p, miR-542-3p/5p, and their counterparts have been associated with the modulation of muscle quality as it pertains to the progression of chronic obstructive pulmonary disease^13,14^. Investigations have underscored alterations in miRNA expression within murine skeletal muscle subjected to hypoxic conditions, pinpointing their involvement in the regulation of AMPK-mediated energy metabolism^15^. However, a notable gap in the current body of research pertains to the paucity of knowledge concerning alterations in the miRNA expression profile during hypoxia-induced skeletal muscle atrophy. Several studies have reported that resistance training may elicit alterations in the expression of miRNAs within skeletal muscle^16–18^. Particular miRNAs, including miR-1-3p, miR-19b-3p, miR-92a, and others, are believed to participate in the regulatory mechanisms through which resistance training ameliorates age-related muscle atrophy^19^. Despite this, no investigations have yet documented the shifts in miRNA expression during hypoxia-induced skeletal muscle atrophy or the potential mitigation of such atrophy through resistance training. Nevertheless, considering the biological processes under miRNA regulation, it is conceivable that miRNAs might function as intermediary regulatory factors influencing the impacts of hypoxia and resistance training on differential gene expression within skeletal muscle.

Therefore, this study initially established a rat model of hypoxia-induced skeletal muscle atrophy, attenuated by resistance training, followed by the screening of differentially expressed miRNAs in skeletal muscle. Subsequently, bioinformatics analysis methods were employed to analyze the potential biological functions of these differentially expressed miRNAs, with the aim of providing a foundation for further investigation into the molecular mechanisms underlying hypoxia-induced skeletal muscle atrophy and the mitigation of muscle wasting by resistance training in the future.

## Results

### Animal model of hypoxia-induced skeletal muscle atrophy and resistance training regimen to mitigate muscle atrophy

**Figure 1:**
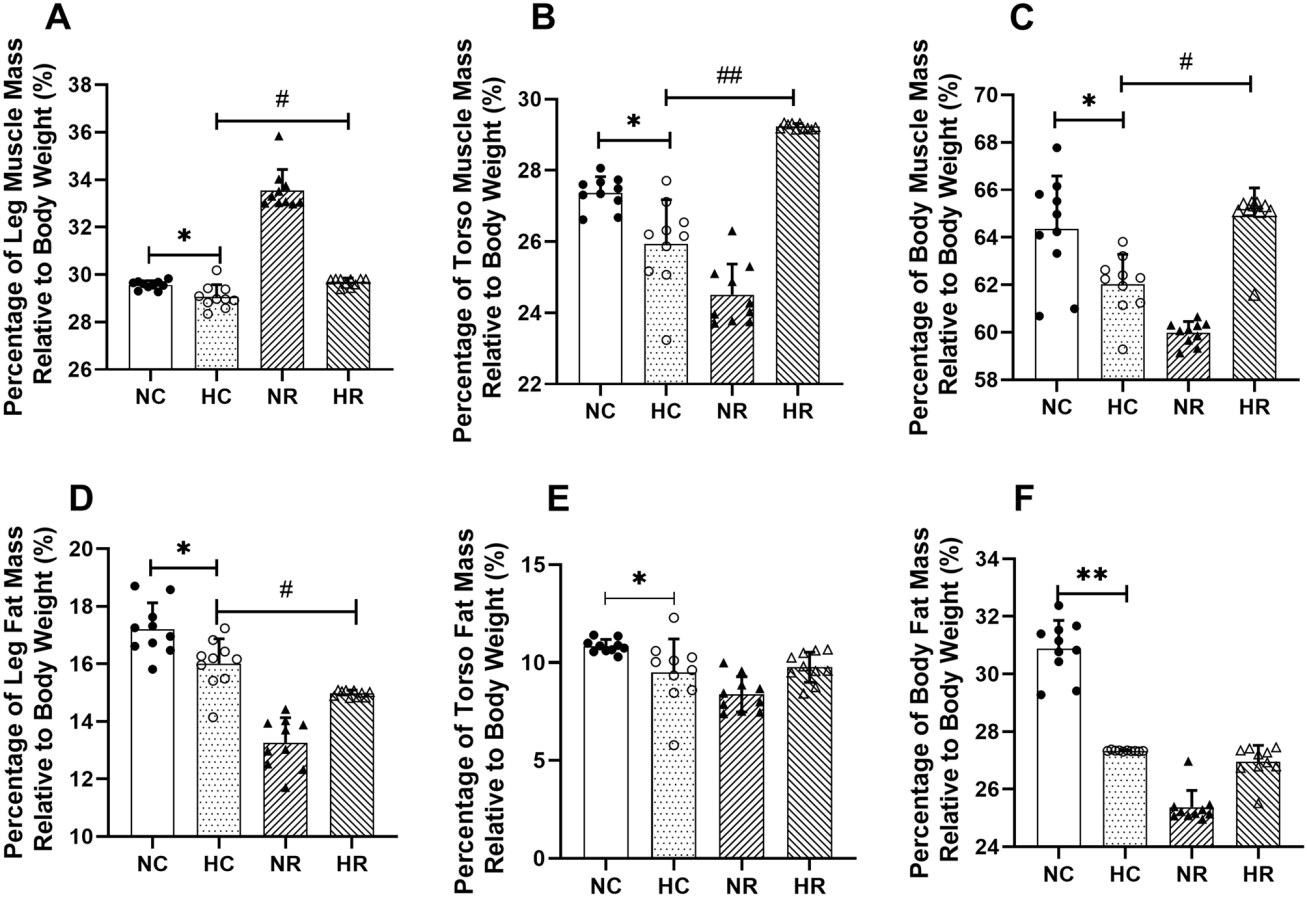
Changes in Rat Body Composition After Intervention (* and ** indicate p < 0.05 and *p* < 0.01 compared to the NC group, respectively; # and ## indicate *p* < 0.05 and *p* < 0.01 compared to the HC group, respectively).

The findings from the body composition analysis revealed notable disparities among the rat groups. Specifically, in the hypoxia control group (HC), distinct reductions were observed in the overall muscle content (12.49%), trunk muscle content (14.32%), and leg muscle content (10.06%), all of which were statistically significant when compared to the normoxia control group (NC) (*p* < 0.05). Conversely, within the hypoxia resistance training group (HR), there were substantial elevations noted in both overall muscle content (15.33%) and leg muscle content (24.42%) in comparison to the hypoxia control group (HC), with statistical significance achieved (*p* < 0.05). Comparing the hypoxia control group (HC) with the normoxia control group (NC), a substantial decrease of 19.35% in overall fat content (*p* < 0.01) and a significant reduction of 15.75% in leg fat content (*p* < 0.05) were evident, while no significant alteration emerged in trunk fat content (*p* > 0.05). It is noteworthy that the hypoxia resistance training group (HR) exhibited no significant changes in overall fat content, trunk fat content, or leg fat content when compared to the hypoxia control group (HC) (*p* > 0.05) (Fig. 1).

**Figure 2:**
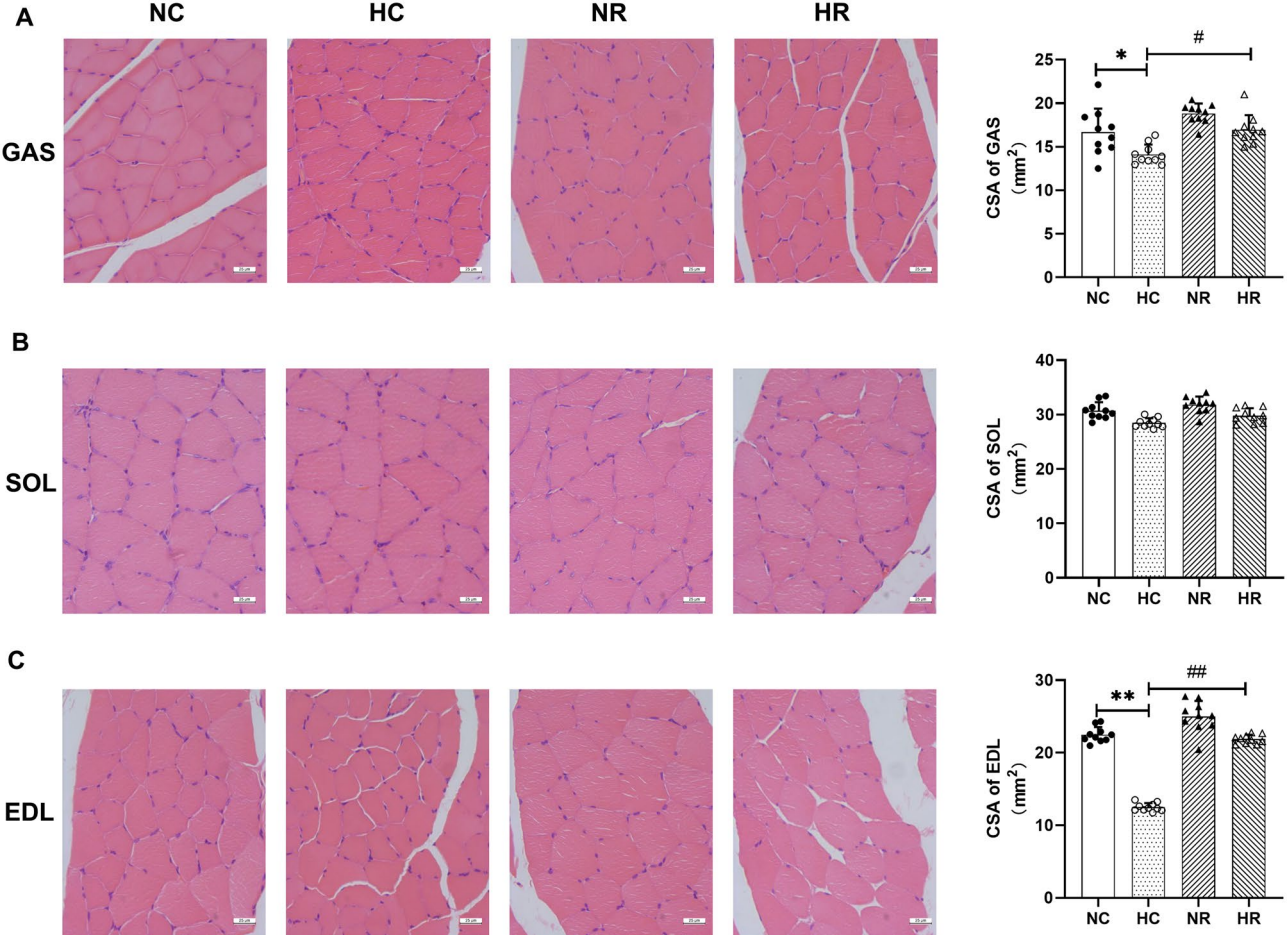
Cross-Sectional Area of Gastrocnemius Muscle, Soleus Muscle, and Extensor Digitorum Longus Muscle in Different Groups. (**A**) HE staining and cross-sectional area of muscle fibers in GAS. (**B**) HE staining and cross-sectional area (CSA) of muscle fibers in the SOL. (C) HE staining and cross-sectional area of muscle fibers in EDL (GAS: gastrocnemius muscle, SOL: soleus muscle, EDL: extensor digitorum longus muscle. Scale bars = 25 µm. * and ** indicate *p* < 0.05 and *p* < 0.01 compared to the NC group, respectively; # and ## indicate *p* < 0.05 and *p* < 0.01 compared to the HC group, respectively).

In comparison to the NC group, the gastrocnemius muscle and extensor digitorum longus muscle in the HC group of rats exhibited a considerable reduction in cross-sectional area (*p* < 0.05), whereas no significant difference was observed in the soleus muscle (*p* > 0.05). Conversely, when juxtaposed with the HC group, the HR group of rats displayed a substantial increase in the cross-sectional area of both the gastrocnemius muscle and extensor digitorum longus muscle (*p* < 0.01), while no significant disparity was detected in the soleus muscle (*p* > 0.05) (Fig. 2).

### The expression profiles of miRNAs in the models of hypoxia-induced skeletal muscle atrophy and resistance training-mediated attenuation of atrophy

**Figure 3:**
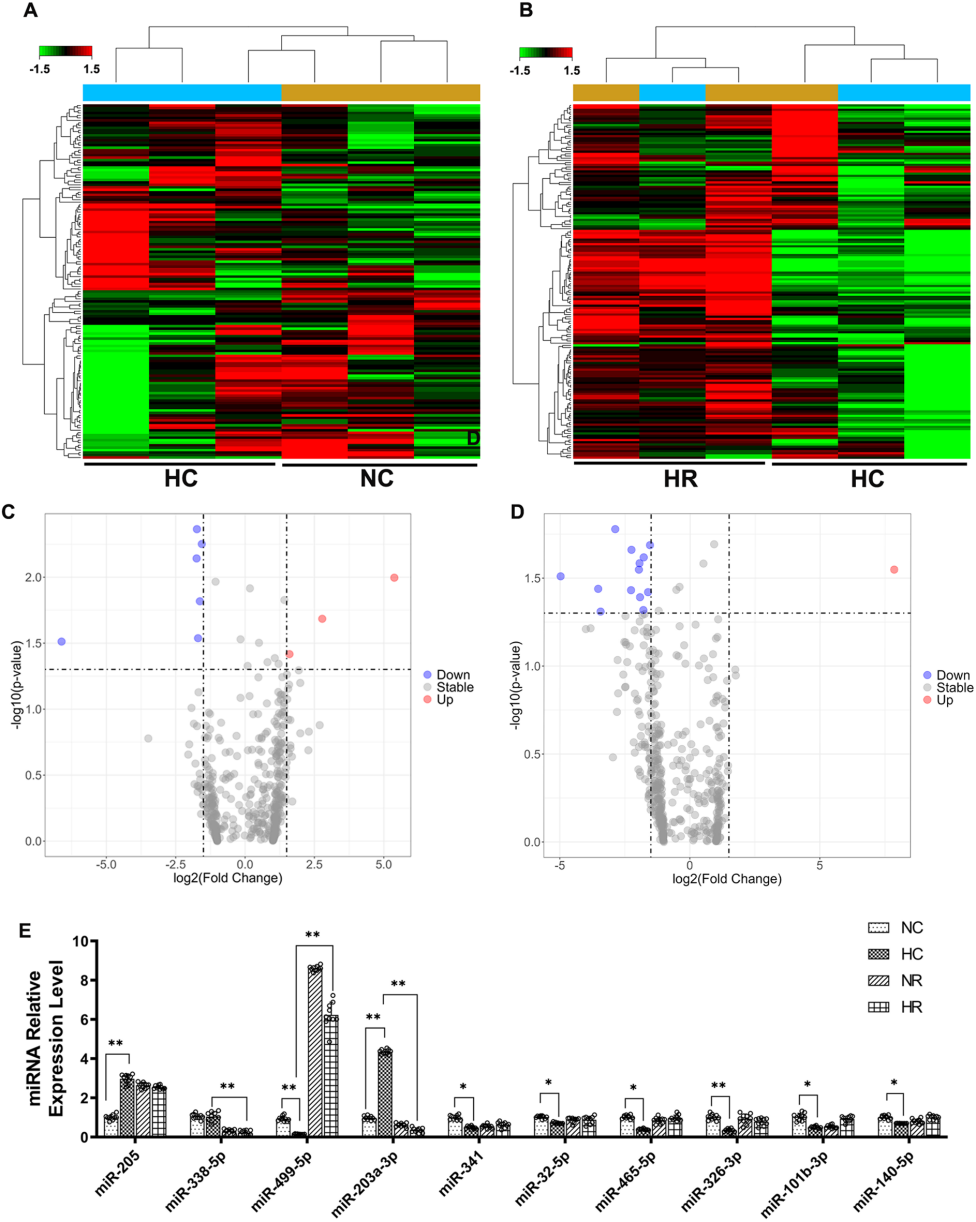
Hierarchical Clustering of Differentially Expressed miRNAs in Rat Skeletal Muscle (Gastrocnemius Muscle) Among Different Groups. (**A**) Cluster heatmap of differentially expressed miRNAs under hypoxic intervention (n = 3). (**B**) Cluster heatmap of dif-ferentially expressed miRNAs under hypoxic resistance training intervention (n = 3). (**C**) Volcano plot of differentially expressed miRNAs under hypoxic intervention. (**D**) Volcano plot of differentially expressed miRNAs under hypoxia-induced resistance training. (n = 3). (**E**) Validation of microarray results by RT-PCR (n = 10). * and ** indicate *p* < 0.05 and *p* < 0.01 between groups, respectively.

459 miRNAs were detected in this study. The results from the miRNA microarray analysis revealed a total of 9 miRNAs exhibiting differential expression (|log2FC|≥ 1.5, *p* < 0.05) between the hypoxia control group (HC) and the normoxia control group (NC). Within this set, 3 miRNAs demonstrated upregulation, while 6 miRNAs displayed downregulation. In comparison, between the hypoxia resistance training group (HR) and the hypoxia control group (HC), a total of 14 miRNAs exhibited differential expression (|log2FC|≥ 1.5, *p* < 0.05), with 1 miRNA showing upregulation and 13 miRNAs undergoing downregulation (Fig. 3, Table 1). This study employed RT-PCR to assess the relative expression levels of 10 miRNAs, including miR-205, miR-338-5p, miR-499-5p, miR-203a-3p, miR-341, miR-32-5p, miR-465-5p, miR-326-3p, miR-101b-3p, and miR-140-5p, in the gastrocnemius muscle. This validation aimed to confirm the microarray results, and the RT-PCR results demonstrated a consistent expression trend with the microarray data (Fig. 3).

**Table 1:** Differentially Expressed miRNAs in Rat Skeletal Muscle.

Group	Transcript ID	Log2(FC)	*p* value	miRNA feature
HC vs. NC	miR-341	− 1.75	7.21E-03	Down
	miR-32-5p	− 1.73	4.33E-03	Down
	miR-465-5p	− 1.56	5.61E-03	Down
	miR-326-3p	− 1.63	1.52E-02	Down
	miR-499-5p	− 6.61	3.08E-02	Down
	miR-760-3p	− 1.69	2.90E-02	Down
	miR-203a-3p	5.38	1.01E-02	Up
	miR-205	2.78	2.07E-02	Up
	miR-1306-3p	1.60	3.83E-02	Up
HR vs. HC	miR-338-5p	− 4.98	3.09E-02	Down
	miR-203a-3p	− 3.54	3.64E-02	Down
	miR-92b-3p	− 2.88	1.67E-02	Down
	miR-199a-5p	− 2.27	3.71E-02	Down
	miR-152-3p	− 2.26	2.18E-02	Down
	miR-497-5p	− 1.97	2.83E-02	Down
	miR-499-5p	7.85	2.83E-02	Up
	miR-130a-3p	− 1.95	2.61E-02	Down
	miR-143-3p	− 1.92	4.06E-02	Down
	miR-101b-3p	− 1.78	2.41E-02	Down
	miR-222-3p	− 1.62	3.80E-02	Down
	miR-139-3p	− 1.54	2.06E-02	Down
	miR-140-5p	− 3.44	4.90E-02	Down
	miR-146b-5p	− 1.80	4.81E-02	Down

### Bioinformatics analysis of differentially expressed miRNAs

By employing the miRWalk, miRanda, and TargetScan databases for target gene prediction and taking into account the convergence of outcomes across these three databases, a comprehensive compilation of 8,298 target genes emerged for the miRNAs exhibiting differential expression due to the influence of hypoxia on skeletal muscle. Similarly, in the context of hypoxia, the miRNAs displaying altered expression as a consequence of resistance training in skeletal muscle resulted in the projection of 14,275 target genes (Table 2).

**Table 2:** Predicted Target Genes of Differentially Expressed miRNAs in Skeletal Muscle.

Group	miRNA ID	Number	Target genes (list of 10 target genes)
HC vs. NC	miR-1306-3p	175	Adra2b, Nras, Cyp2a2, Mobp, Adra2a, Acvrl1, Mapk10, Tnfrsf11b, F2r, Utrn
	miR-203a-3p	1418	Atp2b2, Bdnf, Cyp4a2, Edn1, Eno2, Fgg, Foxg1, Gad1, Gls, Hsd3b5
	miR-205	929	Acp2, B2m, Cat, Cbs, Fancc, Gls, H1f0, Slco2a1, Ngfr, Serpine1
	miR-32-5p	626	Acadm, Ddc, Foxg1, Grm6, Hbb, Ibsp, Nefh, Nefm, Pgam1, Pklr
	miR-326-3p	1730	Add2, Adra2b, Ak1, Atp2b2, Atp4b, Atp7b, Chrm3, Cyp11b2, Cyp21a1, Drd2
	miR-341	21	Slc9a2, Slc30a2, Slc23a2, Kcnd2, Efna2, Pvrl1, Phyhip, Slc25a46, Rtfdc1, Zap70
	miR-465-5p	1035	Acp2, Agtr1a, Atp1a2, Atp1a3, Cd53, Foxg1, Gfi1, Gls, Gpx1, Grm1
	miR-499-5p	796	Ak2, Cat, Cel, Psg19, Ddc, Ednra, Gls, Slco2a1, Cd200, Ppp3ca
	miR-760-3p	1568	A2m, Acly, Add2, Atp2b2, Atp4b, Ddc, Edn1, Ephb1, Ets1, Grm1
HR vs. HC	miR-101b-3p	881	Acp2, Adra2b, Agtr1a, Chrm3, Comt, Ednra, Gad1, Gfap, Gja1, Glra2
	miR-130a-3p	984	Adcyap1r1, Apeh, Atp2b2, Casr, Cyp4a2, Ets1, Gck, Gja1, Gls, Nr3c1
	miR-139-3p	337	Agt, Aldh1a1, B4galt1, Inha, Mbl1, Mx1, Sp1, Tacr1, Tgfa, Tacr2
	miR-140-5p	832	Acp2, Aldh1a1, C2, Cacna1c, Calca, Cyp11b2, Gfap, Glud1, Il1b, Ldha
	miR-143-3p	1014	Adh1, Ak1, Ak2, Alb, Aldoc, Atp1a2, Cad, Eno2, Gck, Gls
	miR-146b-5p	941	Add2, Atp1a2, Atp1b2, Atp7b, Cyp2b1, Cyp4a2, Ddc, Gad2, B4galt1, Grin2b
	miR-152-3p	1325	Aldoc, Akr1b1, Atp1a2, Atp1b2, Tspo, Casr, Cat, Chrm3, Cp, Epo
	miR-199a-5p	1287	Acp1, Acp2, Agt, Agtr1a, Atp2b2, Atp4b, Chrm3, Comt, Cyp1a1, Cyp21a1
	miR-203a-3p	1418	Atp2b2, Bdnf, Cyp4a2, Edn1, Eno2, Fgg, Foxg1, Gad1, Gls, Hsd3b5
	miR-222-3p	751	Add2, Agtr1a, Ak2, Aldh1a1, Atp1a1, Psg19, Ddc, Ets1, Gja1, Grm2
	miR-338-5p	1039	Acp1, Adcyap1, Atp1b2, Cyp1a2, Ets1, Gls, Hprt1, Il1b, Slco2a1, Pkib
	miR-497-5p	2042	Acadm, Add2, Adrb2, Atp2b2, Atp7b, Bdnf, Camk2b, Cd53, Cel, Chga
	miR-499-5p	796	Ak2, Cat, Cel, Psg19, Ddc, Ednra, Gls, Slco2a1, Cd200, Ppp3ca
	miR-92b-3p	628	Acadm, Ddc, Foxg1, Grm6, Hbb, Ibsp, Nefh, Nefm, Pgam1, Pklr

GO analysis revealed that among the differentially expressed miRNAs between HC and NC, increased miRNAs were enriched in biological processes such as small GTPase-mediated signal transduction, positive regulation of cell migration, and regulation of transcription DNA-dependent, totaling 686 terms. Increased miRNAs were also enriched in molecular functions including protein binding, DNA binding, and zinc ion binding, with a total of 205 terms. Additionally, increased miRNAs were enriched in cellular components such as cytoplasm, nucleus, and nucleolus, totaling 169 terms. On the other hand, decreased miRNAs were enriched in biological processes such as positive regulation of cell migration, positive regulation of transcription DNA-dependent, and positive regulation of transcription from RNA polymerase II promoter, totaling 942 terms. They were also enriched in molecular functions including protein binding, zinc ion binding, and molecular_function, totaling 277 terms. Furthermore, decreased miRNAs were enriched in cellular components such as nucleus, plasma membrane, and membrane, totaling 217 terms.

**Figure 4:**
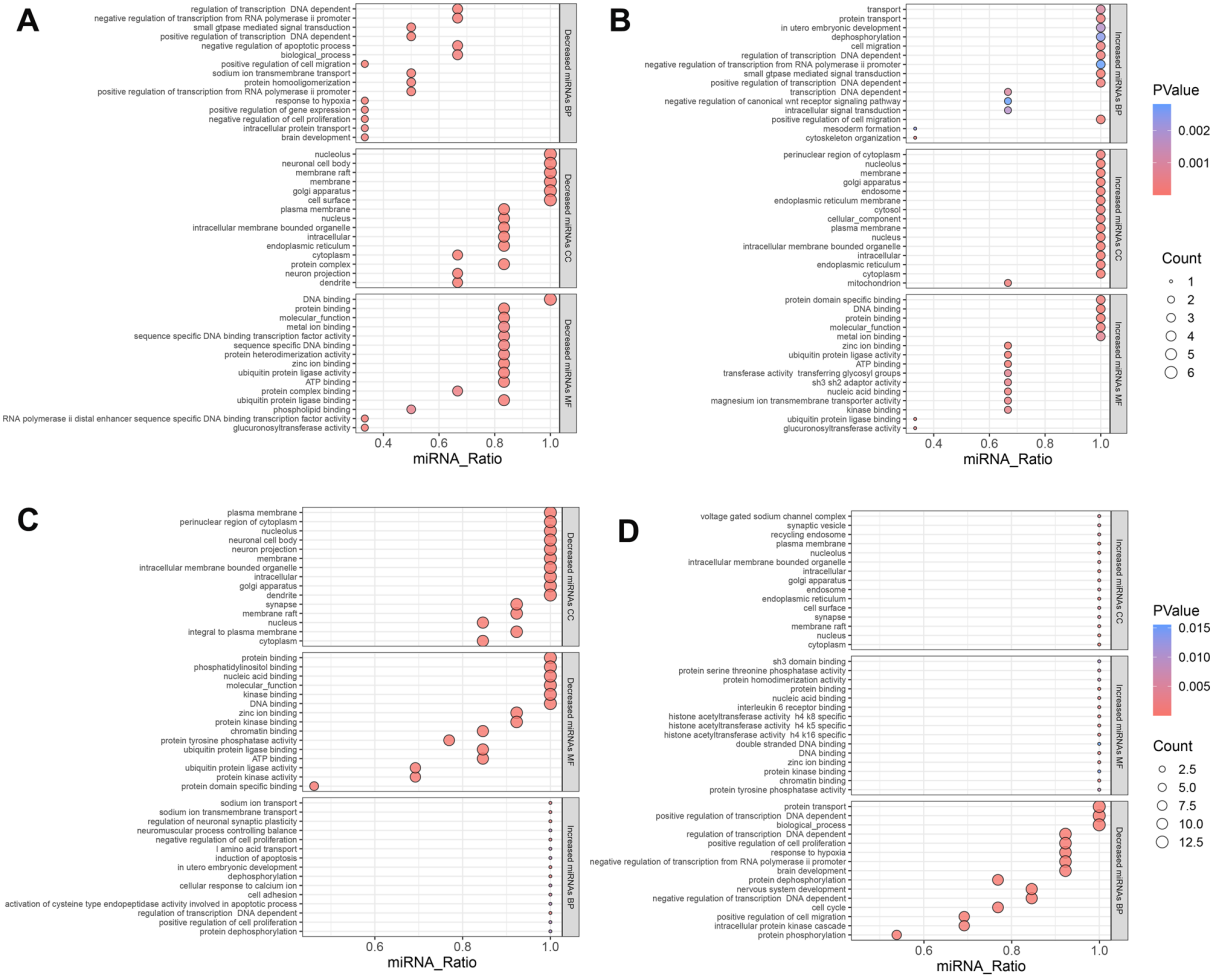
GO Analysis of Differentially Expressed miRNAs in Skeletal Muscle. (**A**) GO analysis of down-regulated miRNA expression af-fected by hypoxia. (**B**) GO analysis of up-regulated miRNA expression affected by hypoxia. (**C**) GO analysis of down-regulated miRNAs affected by resistance training under hypoxia. (**D**) GO analysis of up-regulated miRNAs affected by resistance training under hypoxia.

Regarding the differentially expressed miRNAs between HR and HC, increased miRNAs were enriched in biological processes such as regulation of neuronal synaptic plasticity, regulation of transcription DNA-dependent, and dephosphorylation, totaling 197 terms. They were also enriched in molecular functions including protein binding, histone acetyltransferase activity H4 K16 specific, and histone acetyltransferase activity H4 K8 specific, totaling 68 terms. Moreover, increased miRNAs were enriched in cellular components such as nucleus, cytoplasm, and nucleolus, totaling 76 terms. Conversely, decreased miRNAs were enriched in biological processes such as regulation of transcription DNA-dependent, negative regulation of transcription DNA-dependent, and nervous system development, totaling 1416 terms. They were also enriched in molecular functions including zinc ion binding, protein binding, and DNA binding, totaling 413 terms. Additionally, decreased miRNAs were enriched in cellular components such as cytoplasm, nucleus, and Golgi apparatus, totaling 296 terms. The top 15 enriched terms are presented in the Fig. 4.

**Figure 5:**
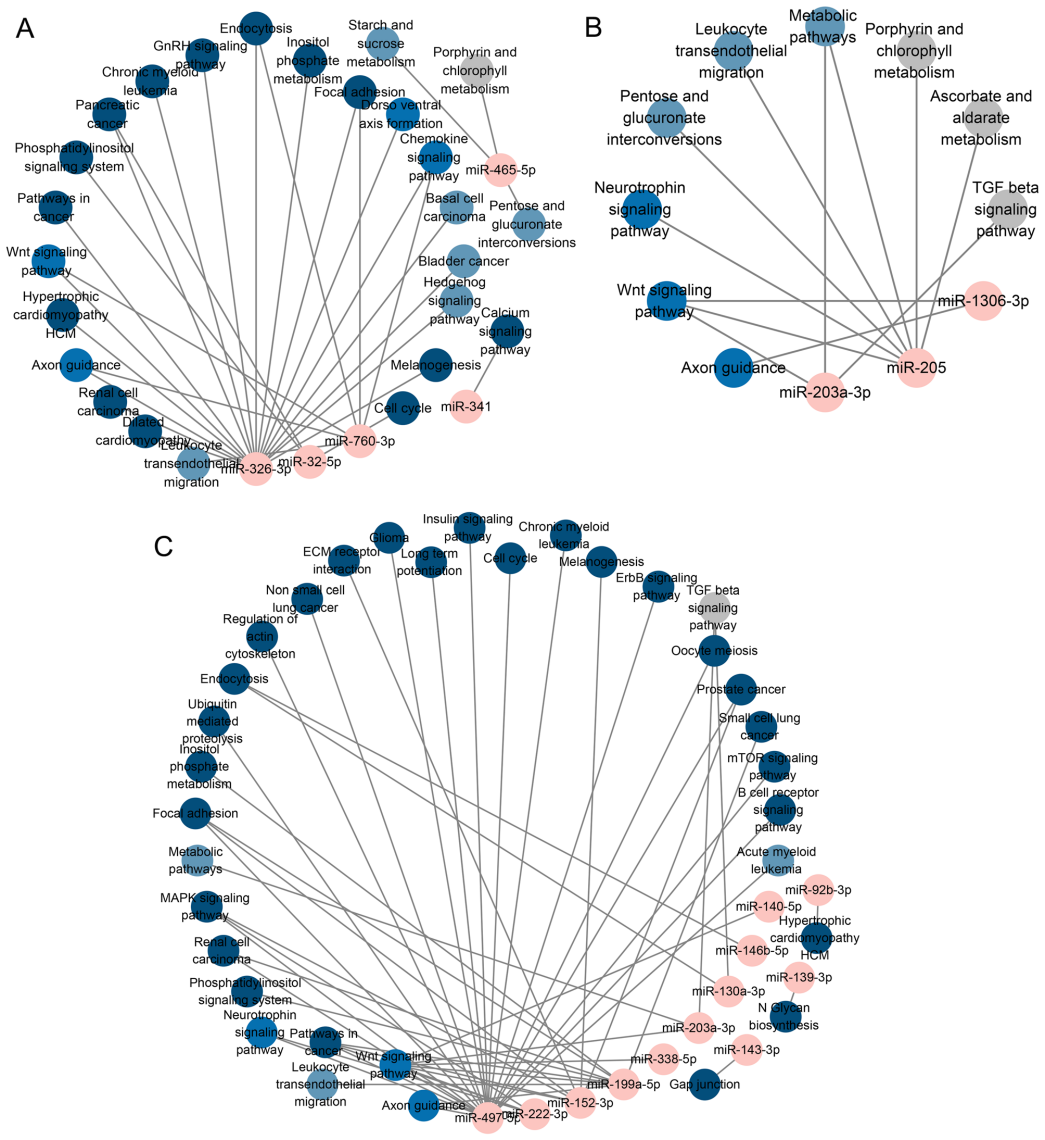
Regulatory Signaling Pathway Network Governed by Differentially Expressed miRNAs. (**A**) Regulatory signaling pathway net-work affected by hypoxia down-regulated miRNAs. (**B**) Regulatory signaling pathway network affected by hypoxia up-regulated miRNAs. (**C**) Regulatory signaling pathway network affected by resistance training down-regulated miRNAs under hypoxia.

The KEGG pathway analysis revealed that among the differentially expressed miRNAs between HC and NC, increased miRNAs were enriched in pathways such as Metabolic pathways, Pentose and glucuronate interconversions, and Leukocyte transendothelial migration, totaling 9 pathways. On the other hand, decreased miRNAs were enriched in pathways including the Wnt signaling pathway, Pathways in cancer, and Axon guidance, totaling 25 pathways. In the comparison between HR and HC, increased miRNAs did not exhibit enrichment in any specific pathways. However, decreased miRNAs were enriched in pathways such as the Wnt signaling pathway, Axon guidance, and Focal adhesion, totaling 33 pathways (Fig. 5). All GO and KEGG analysis results can be found in Supplementary Information 1–8.

## Discussion

This study observed a decrease in skeletal muscle mass and reduced muscle fiber cross-sectional area in rats exposed to hypoxic conditions. Resistance training demonstrated some improvement in hypoxia-induced muscle atrophy. Hypoxia resulted in differential expression of 9 miRNAs in skeletal muscle (miR-341, miR-32-5p, miR-465-5p, miR-326-3p, miR-499-5p, miR-760-3p, miR-203a-3p, miR-205, and miR-1306-3p), while resistance training under hypoxic conditions induced differential expression of 14 miRNAs in skeletal muscle (miR-338-5p, miR-203a-3p, miR-92b-3p, miR-199a-5p, miR-152-3p, miR-497-5p, miR-499-5p, miR-130a-3p, miR-143-3p, miR-101b-3p, miR-222-3p, miR-139-3p, miR-140-5p, and miR-146b-5p). Bioinformatics analysis indicates that differentially expressed miRNAs play a regulatory role in the progression of hypoxia-induced muscle atrophy in rats and in improving muscle atrophy through resistance training, potentially acting through multiple signaling pathways.

The hypoxic environment induced skeletal muscle atrophy in this study, manifested by decreased muscle mass and reduced muscle fiber cross-sectional area, consistent with findings from most previous studies. In conditions of low pressure and hypoxia, rats tend to exhibit slower weight gain. After a 5-week intervention in a simulated low-pressure and low-oxygen environment, akin to an altitude of 6000 m, the body weight, wet weight of the gastrocnemius muscle, and muscle fiber cross-sectional area of rats mirrored those of rats kept under normoxic conditions for 3 weeks^20^. In another study conducted in a decompression chamber to simulate low-pressure and low-oxygen conditions equivalent to an altitude of 4000 m (463 torr), it was noted that rats exposed to hypoxia for 12 weeks underwent a significant decrease in body weight. The soleus muscle and extensor digitorum longus muscle similarly demonstrated marked reductions in size. Moreover, this investigation established that the cross-sectional area of muscle fibers in the soleus muscle and extensor digitorum longus muscle decreased by 22% and 32%, respectively^21^. During a 5-week intervention in a decompression chamber simulating a low-oxygen environment equivalent to an altitude of 5000 m, a noteworthy reduction in the cross-sectional area of gastrocnemius muscle fibers was observed in rats. Remarkably, this reduction occurred concurrently in both types of muscle fibers^22^.

Under hypoxic conditions, resistance training in this study ameliorated hypoxia-induced skeletal muscle atrophy, indicating that appropriate protocols can promote muscle hypertrophy in such environments. Previous research has reported significant improvements in body weight and lean body mass before and after intervention with resistance training under normobaric hypoxia (14.4% oxygen concentration), showing a 6.1% increase in muscle cross-sectional area of the quadriceps, biceps, and hamstrings^23^. Similarly, exercise under both normoxic (FiO2 = 20.9%) and hypoxic (FiO2 = 12.7%) conditions demonstrated that resistance training significantly increased the thickness of the biceps and triceps brachii, stimulating skeletal muscle hypertrophy^24^. However, in this study, the muscle mass of the torso and whole body relative to body weight did not increase, possibly due to the training protocol employed. Specifically, the resistance training involved climbing, which primarily focused on training the muscles of the limbs, resulting in relatively less stimulation of the muscles in the torso. This could also be one of the reasons for the less noticeable change in torso muscle mass.

Within the scope of this study, hypoxia precipitated the differential expression of 9 miRNAs. Some of these miRNAs have previously been linked to hypoxia stimulation or implicated in muscle atrophy. For instance, In rat models exposed to hypoxic conditions (9–11% oxygen concentration for 8 h daily over 4 weeks), miR-499 expression was noted to decrease in the gastrocnemius and biceps femoris muscles^25^. MiR-760-3p involvement in hypoxia-induced cell proliferation and apoptosis has been documented, with its expression diminished in hypoxia-exposed human pulmonary artery smooth muscle cells^26^. Further down the spectrum, miR-203a-3p's role appears tied to skeletal muscle differentiation. In zebrafish embryos, it regulates skeletal muscle differentiation by targeting *Dmrt2a*^27^. Notably, in a rat model simulating an altitude of 6000 m, miR-203a-3p expression in lung tissue was significantly downregulated after 72 h of intervention^28^. Similarly, within a rat ischemic injury model, miR-205 exhibited significant increases in skeletal muscle expression after ischemic periods and reperfusion^29^. The remaining four differentially expressed miRNAs in this study—miR-341, miR-465-5p, miR-326-3p, and miR-1306-3p—have yet to be linked to low oxygen intervention or muscle atrophy. Their precise roles in mediating low oxygen-induced skeletal muscle atrophy require further exploration.

In hypoxic conditions, resistance training induced distinctive miRNA expression patterns in skeletal muscle, involving 14 miRNAs. Among this cohort of miRNAs, studies have illuminated the role of miR-199a-5p in Duchenne muscular dystrophy (DMD), where it influences the WNT signaling pathway via genes such as *FZD4*, *JAG1*, and *WNT2*, thereby impacting cellular proliferation and myogenic differentiation. Elevated miR-199a-5p expression has also been linked to aberrant muscle fiber degradation^30^. MiR-143-3p upregulation in the blood of amyotrophic lateral sclerosis (ALS) patients marks it as a potential miRNA biomarker for muscle atrophy^31^. miR-146b-5p has emerged as a pivotal player; its elevated expression in skeletal muscle samples of type 2 myotonic dystrophy (DM2) patients suggests its potential as a miRNA biomarker for muscle atrophy^32^. In the context of miR-222-3p and skeletal muscle, its role extends to energy metabolism^33^. Additional miRNAs, including miR-92b-3p, miR-152-3p, and miR-140-5p, have received less attention in the context of skeletal muscle growth, development, and physiology.

Virtually all genes hold the potential to be under miRNA regulation, with each miRNA capable of targeting hundreds of genes^34^. Throughout gene expression, miRNAs have the ability to curtail mRNA transcription into proteins. This is achieved by targeting the 3’UTR of genes. Additionally, miRNAs can bind to mRNA, thereby inducing mRNA degradation, culminating in a reduction in gene expression levels. Upon delving into the predicted target genes, it becomes evident that the miRNAs are differentially expressed due to hypoxia, incline target genes linked to atrophy. For example, *Utrn* (Utrophin) is one of the target genes of miR-1306-3p. This gene, akin in structure and function to the dystrophin gene (which encodes the anti-atrophy protein), is also recognized as a dystrophin-associated protein. Notably, it contributes to the regulatory machinery governing certain facets of atrophy^35,36^. Murine double minute 2 (*Mdm2*) has emerged as a direct target gene of miR-205. This gene encodes an E3 ubiquitin ligase that resides within the nucleus. Interestingly, *Mdm2* showcases heightened expression within a hindlimb suspension-induced muscle atrophy model^37^. Furthermore, *Ube1*,* Rbx1*, and other elements also count among the target genes influenced by miR-205. These genes actively participate in the ubiquitin-mediated protein degradation process^38,39^, which potentially extends to the intricate regulation of skeletal muscle mass. Beyond the aforementioned miRNAs, various other differentially expressed miRNAs are likewise associated with target genes central to muscle atrophy regulation. Notably, miR-326-3p target genes include *Stk11*, *Ikbkb*, and *Pi3k*, while miR-465-5p target genes include *Aire*, *Apc8*, and *Rbx1*. These miRNAs could influence muscle atrophy by overseeing the degradation of skeletal muscle proteins.

In the KEGG pathway analysis, it is evident that upregulated miRNAs and downregulated miRNAs target different signaling pathways. It is noteworthy that within these pathways, the upregulated miRNAs target the Wnt signaling pathway, MAPK-TGF beta signaling pathway, while the downregulated miRNAs target the Wnt signaling pathway and GnRH signaling pathway. Additionally, hypoxia-induced resistance training may target signaling pathways such as the Wnt signaling pathway, Insulin signaling pathway, MAPK signaling pathway, TGF beta signaling pathway, Ubiquitin-mediated proteolysis, and mTOR signaling pathway, which have been reported to potentially influence skeletal muscle quality. In the current study, bioinformatics predictions pinpointed 7 miRNAs, including miR-1306-3p, miR-203a-3p, and miR-205, as potential regulators of the Wnt signaling pathway. This points toward their plausible role as regulatory factors implicated in the low oxygen-induced effects on skeletal muscle quality. Studies reported that under hypoxic stimulation, HIF1α negatively regulates skeletal muscle regeneration by inhibiting the classical Wnt signaling pathway^40^. The skeletal muscle TGF-β signaling pathway holds sway over muscle growth and development. TGF-β exerts its regulatory role in muscle atrophy by activating both the classical Smad-dependent pathway and nonclassical routes, such as ERK1/2, JNK1/2, and p38 MAPK. The engagement of Smad3, ERK1/2, and JNK1/2 is integral to TGF-β's capacity to induce muscle atrophy^41^. In a mouse model of skeletal muscle atrophy induced by amyotrophic lateral sclerosis (ALS) (hSOD1G93A mice), the mRNA levels of TGF-β1, total Smad3 protein levels, and p-Smad3 displayed marked increases^42^. Similarly, augmented TFGβ signaling has been discerned in congenital myopathies^43^. Transcriptional and methylation analyses of individuals undergoing acute and chronic resistance exercise interventions revealed that the manipulation of three genes, *FOS*, *SMAD3*, and *WNT9A*, within the TGF-β signaling pathway impacted skeletal muscle synthesis, metabolism, and hypertrophy in response to resistance training^44^. In a rat resistance training model, the TGF-β1-Smad signaling pathway mediated the muscle hypertrophy elicited by resistance training. This mechanism encompasses a substantial reduction in skeletal muscle TGF-β1 expression and phosphorylation of Smad3 COOH-terminal residues (P-Smad3 S423/425) following resistance training. This results in heightened phosphorylation levels of SOH2 (P-Smad2-LS245/250/255) and Smad3 (P-Smad3-LSer208), culminating in a diminished inhibition of satellite cell activation and protein synthesis, ultimately fostering hypertrophy^45^. Furthermore, differentially expressed miRNAs can also target the ubiquitin-mediated protein degradation signaling pathway (such as miR-203a-3p, miR-205, and miR-32-5p) and the mTOR signaling pathway (such as miR-32-5p, miR-326-3p, and miR-760-3p).

This study also has some limitations. Firstly, the utilization of microarray technology in our investigation presents inherent biases and limitations compared to RNAseq methods. While microarray analysis allows for comprehensive profiling of miRNA expression, it is acknowledged that RNAseq possesses certain advantages such as improved sensitivity and the ability to detect novel transcripts. We recognize the necessity to elucidate these methodological differences and their potential impacts on our findings. Moreover, an important consideration pertains to the translatability of our results from rodent models to human physiology. Although our study extensively discusses mechanisms and pathways observed in rodent models, the direct extrapolation of these findings to human biology requires cautious interpretation. We acknowledge the complexities inherent in inter-species variations and biological contexts. Exploring these distinctions in detail is essential to accurately evaluate the significance and applicability of our findings to human conditions. Finally, experimental validation is necessary to confirm the roles of signaling pathways identified by bioinformatics in hypoxia-induced muscle atrophy.

## Conclusions

Resistance training has shown potential in ameliorating hypoxia-induced skeletal muscle atrophy in rats, with miRNAs emerging as key regulators in this context. Differentially expressed miRNAs exert regulatory influence over both the progression of hypoxia-induced muscle atrophy and its amelioration through resistance training in rats, potentially operating through a multitude of signaling pathways.

## Materials and methods

### Animals and grouping

This study was performed in accordance with relevant guidelines and regulations. Animal experiments were carried out in accordance with the ARRIVE guidelines and approved by the Ethics Committee for Sports Science Experiments at Beijing Sport University (approval number: 2023043A). Male Sprague Dawley (SD) rats, aged eight weeks, were procured from Beijing Vital River Laboratory Animal Technology Co., Ltd. Following a one-week acclimatization period, body composition was evaluated using dual-energy X-ray absorptiometry (DEXA). Subsequently, the rats were allocated randomly into four groups, each comprising 10 rats: the normoxic control group (NC), the normoxic resistance exercise group (NR), the hypoxic control group (HC), and the hypoxic resistance exercise group (HR). The rats were individually housed in cages under tightly controlled environmental conditions, maintaining a temperature range of 22–25 °C, relative humidity of 50–80%, and a 12-h light cycle from 7:00 AM to 7:00 PM. They were provided with standard rodent maintenance feed and had unrestricted access to food. Daily recordings of body weight and food intake were recorded.

### Intervention protocol and sample collection

Following a week of adaptive training, the formal experimental intervention commenced. Adaptation phase was conducted under normoxic conditions.During the adaptation phase, rats underwent ladder climbing training without the addition of weights. For the formal intervention, rats in the HC and HR groups were exposed to a controlled oxygen concentration of 11.2%, simulating hypoxic conditions equivalent to an altitude of 4500 m. This hypoxic exposure was maintained for four weeks. In NR and HR, resistance exercise was conducted using a ladder-climbing simulation apparatus, with ladder dimensions measuring 110 cm × 18 cm and a step spacing of 2 cm. The resistance training protocol adhered to previous research practices and included the following steps: starting with a load equivalent to 30% of their body weight for the initial training session, followed by a gradual increase of 15% of their body weight for each subsequent session until reaching a load of 130% of their body weight. This load was then maintained for the remainder of the intervention period. Training sessions occurred every alternate day, comprising three sets of five repetitions per session, with a rest interval of 3–5 min between sets. This training regimen was conducted consistently over four weeks. The rats in the NR group underwent resistance training in a normoxic environment, while those in the HR group underwent resistance training in a hypoxic environment (oxygen concentration of 11.2%).

Sample collection occurred 72 h after the intervention. Rats were humanely euthanized under anesthesia, and blood was drawn from the abdominal aorta. Skeletal muscles, including the gastrocnemius (GAS), extensor digitorum longus (EDL), and soleus (SOL), were meticulously isolated. Muscle samples were cleansed in physiological saline to eliminate residual blood, excess moisture was absorbed with filter paper, and the weight of wet muscle was measured. Skeletal muscle tissues were then segregated and treated as follows: muscles designated for histological examination were fixed in a 4% paraformaldehyde solution; muscles intended for microarray analysis were preserved in RNAlater solution, initially stored at 4 °C, then shifted to -20 °C, and finally transferred to an ultralow temperature freezer at − 80 °C.

### Body composition analysis

Rat body composition was assessed using a dual-energy X-ray absorptiometry (DXA) scanner (Lunar iDXA, GE Healthcare, USA) both before the adaptation phase and upon completion of the hypoxic training intervention. Rats were anesthetized with a sodium pentobarbital solution at a concentration of 3 mg/ml, administered at a rate of 1 ml/100 g of body weight. For the groups subjected to hypoxic intervention, anesthesia was administered within a low-oxygen environment, while the remaining rats were anesthetized under normoxic conditions. Body composition measurements were conducted on the anesthetized rats.

### Hematoxylin–eosin staining

Following fixation, skeletal muscle tissue was cut into cubes measuring 0.5 cm × 0.5 cm × 0.5 cm. These cubes were positioned to ensure that the muscle fibers were perpendicular to the section before being embedded. Paraffin sections with a thickness of 5 μm were subsequently prepared. To examine histological alterations in the skeletal muscle tissue, hematoxylin and eosin staining were applied. From each slide, three fields of view were selected, and within each field of view, five images were captured. The cross-sectional area of the skeletal muscle was quantified utilizing ImageJ software.

### RNA isolation and small RNA profiling

RNA extraction was carried out from 50 mg of gastrocnemius muscle samples using the miRNeasy Mini Kit (Qiagen, Germany). The concentration and purity of the extracted RNA were determined using a Nanodrop 2000 spectrophotometer, ensuring an OD260/OD280 ratio within the range of 2.0 to 2.2. A 1.5% formaldehyde denaturing gel electrophoresis was performed at 120 V for 15 min, followed by gel imaging to evaluate the integrity of the 28S and 18S ribosomal RNA bands.

For miRNA expression profiling in the gastrocnemius muscle, the Affymetrix miRNA 4.0 microarray (Affymetrix, USA) was employed. This microarray comprises 30,424 miRNA probe sets, encompassing 728 mature rat miRNAs and 490 pre-miRNAs. The labeling of samples followed the standard procedure (Flash Tag Biotin HSR RNA Labeling Kit, Affymetrix, USA). Subsequently, the samples were subjected to microarray hybridization (GeneChip2 Hybridization Oven 640, Affymetrix, USA), followed by washing (GeneChip2 Fluidics Station 450, USA) and finally scanning (GeneChip2 Scanner 3000 7G, Affymetrix, USA). Signal intensity values were evaluated using Affymetrix Expression Console™ software (version 1.3.1). The experimental outcomes were analyzed and processed, and the sample expression data were subjected to normalization procedures.

#### RT-PCR

The miRcute Plus miRNA qPCR Kit (SYBR Green) (Tiangen, China) was used for detection. The upstream primers for miRNA were as follows: miR-205: CGTCCTTCATTCCACCGG, miR-338-5p: CGCGTGGAGTGTGGAGACT, miR-499-5p: CGCGCTTAAGACTTGCAGTGATGTTT, miR-203a-3p: CGCCGTGAAATGTTTAGGACCACTAG, miR-341: TCGGTCGATCGGTCGGTC, miR-32-5p: CGCGCTATTGCACATTACTAAGTTGCA, miR-465-5p: CGCTATTTAGAACGGTGCTGGTGTG, miR-326-3p: TATACCTCTGGGCCCTTCCTCC, miR-101-3p: GCGCGCGTACAGTACTGTGATA, miR-140-5p: CGCGCAGTGGTTTTACCCTA. The downstream primers used the universal primers provided with the kit. Reaction system (20 µl): 10 µl 2 × miRcute Plus miRNA PreMix (SYBR&ROX), 0.4 µl Forward Primer, 0.4 µl Reverse Primer (10 µM), 1 µl miRNA first-strand cDNA, and 9.2 µl ddH2O. Reaction program: 15 min at 95℃ for one cycle; 20 s at 94 °C, 34 s at 60 °C for 45 cycles; Melting/Dissociation Curve Stage.

### Differential expression miRNA bioinformatics analysis

For predicting target genes, the miRWalk platform (http://mirwalk.umm.uni-heidelberg.de/) was utilized, incorporating three target gene prediction databases: miRWalk, miRanda, and TargetScan. Genes that were consistently predicted by all three target gene databases were chosen as the target genes for subsequent analysis. The miRWalk platform was also utilized for conducting Gene Ontology (GO) enrichment analysis and KEGG pathway analysis of the miRNAs^46–48^. Additionally, Cytoscape version 3.10.0 was employed to construct an interactive regulatory network illustrating the interactions between miRNAs and KEGG signaling pathways.

### Data analysis

The data were analyzed using SPSS v22.0 software (SPSS Inc., Chicago, IL, USA). Results are presented as mean ± standard deviation (mean ± SD). To compare multiple groups, a two-way analysis of variance (ANOVA) was conducted. In case of a significant interaction effect between the two factors, simple effects tests were performed. For post hoc comparisons in the absence of an interaction effect, the Tukey method was employed instead of the least significant difference (LSD) method to minimize potential multiple testing bias. Statistical significance was set at *P* < 0.05. Statistical analysis was performed using a t-test to evaluate the alterations in miRNA expression between the two groups. The *p*-values were adjusted using the Benjamini–Hochberg correction method, with an FDR < 0.05 threshold applied. Additionally, ∣log2(FC)∣ ≥ 1.5 was used as the criterion for filtering differentially expressed miRNAs.

### Institutional review board statement

This study was performed in accordance with relevant guidelines and regulations. Animal experiments were carried out in accordance with the ARRIVE guidelines and approved by the Ethics Committee for Sports Science Experiments at Beijing Sport University (approval number: 2023043A).

### Supplementary Information

Supplementary Information 1.

Supplementary Information 2.

Supplementary Information 3.

Supplementary Information 4.

Supplementary Information 5.

Supplementary Information 6.

Supplementary Information 7.

Supplementary Information 8.

## Data Availability

Data are available on request from the corresponding author.

## References

[1] Fullerton ZS, McNair BD, Marcello NA, Schmitt EE, Bruns DR (2022). Exposure to high altitude promotes loss of muscle mass that is not rescued by metformin. High Alt. Med. Biol..

[2] Son JS (2016). Effect of resistance ladder training on sparc expression in skeletal muscle of hindlimb immobilized rats. Muscle Nerve.

[3] Krug AL (2016). High-intensity resistance training attenuates dexamethasone-induced muscle atrophy. Muscle Nerve.

[4] Yang X (2023). Exercise mitigates Dapagliflozin-induced skeletal muscle atrophy in STZ-induced diabetic rats. Diabetol. Metab. Syndr..

[5] Liu Y (2021). Eight weeks of high-intensity interval static strength training improves skeletal muscle atrophy and motor function in aged rats via the PGC-1alpha/FNDC5/UCP1 Pathway. Clin. Interv. Aging.

[6] Rathor R, Agrawal A, Kumar R, Suryakumar G, Singh SN (2021). Ursolic acid ameliorates hypobaric hypoxia-induced skeletal muscle protein loss via upregulating Akt pathway: An experimental study using rat model. IUBMB Life.

[7] Chaudhary P (2012). Chronic hypobaric hypoxia mediated skeletal muscle atrophy: role of ubiquitin-proteasome pathway and calpains. Mol. Cell. Biochem..

[8] Oikawa S, Akimoto T (2023). Functional analysis of MicroRNAs in skeletal muscle. Methods Mol. Biol..

[9] Sharma M, Juvvuna PK, Kukreti H, McFarlane C (2014). Mega roles of microRNAs in regulation of skeletal muscle health and disease. Front. Physiol..

[10] Shin YJ (2020). A subset of microRNAs in the Dlk1-Dio3 cluster regulates age-associated muscle atrophy by targeting Atrogin-1. J. Cachexia Sarcopenia Muscle.

[11] Itokazu M (2022). Adipose-derived exosomes block muscular stem cell proliferation in aged mouse by delivering miRNA Let-7d-3p that targets transcription factor HMGA2. J. Biol. Chem..

[12] Jung HJ (2017). Comprehensive miRNA profiling of skeletal muscle and serum in induced and normal mouse muscle atrophy during aging. J. Gerontol. A Biol. Sci. Med. Sci..

[13] Garros RF (2017). MicroRNA-542 promotes mitochondrial dysfunction and SMAD activity and is elevated in intensive care unit-acquired weakness. Am. J. Respir. Crit. Care Med..

[14] Connolly M (2018). miR-424-5p reduces ribosomal RNA and protein synthesis in muscle wasting. J. Cachexia Sarcopenia Muscle.

[15] Hadj-Moussa H (2021). MicroRNA-mediated inhibition of AMPK coordinates tissue-specific downregulation of skeletal muscle metabolism in hypoxic naked mole-rats. J. Exp. Biol..

[16] Spakova I (2020). MicroRNA molecules as predictive biomarkers of adaptive responses to strength training and physical inactivity in haemodialysis patients. Sci. Rep..

[17] Domanska-Senderowska D (2019). MicroRNA profile and adaptive response to exercise training: A review. Int. J. Sports Med..

[18] Ogasawara R (2016). MicroRNA expression profiling in skeletal muscle reveals different regulatory patterns in high and low responders to resistance training. Physiol. Genomics.

[19] Rivas DA (2021). miR-19b-3p is associated with a diametric response to resistance exercise in older adults and regulates skeletal muscle anabolism via PTEN inhibition. Am. J. Physiol. Cell Physiol..

[20] Snyder GK, Wilcox EE, Burnham EW (1985). Effects of hypoxia on muscle capillarity in rats. Respir. Physiol..

[21] Bigard AX, Brunet A, Guezennec CY, Monod H (1991). Effects of chronic hypoxia and endurance training on muscle capillarity in rats. Pflugers Arch..

[22] Huang Q (2000). Effects of hypoxia alone or exercise combined on capillarization of rat gastrocnemius muscle and its mechanism. Zhonghua Bing Li Xue Za Zhi.

[23] Kon M (2015). Effects of systemic hypoxia on human muscular adaptations to resistance exercise training. Physiol. Rep..

[24] Kurobe K (2015). Effects of resistance training under hypoxic conditions on muscle hypertrophy and strength. Clin. Physiol. Funct. Imaging.

[25] Huang S (2016). Electrical stimulation influences chronic intermittent hypoxia-hypercapnia induction of muscle fibre transformation by regulating the microRNA/Sox6 pathway. Sci. Rep..

[26] Yang YZ (2018). miR-760 mediates hypoxia-induced proliferation and apoptosis of human pulmonary artery smooth muscle cells via targeting TLR4. Int. J. Mol. Med..

[27] Lu C (2017). MicroRNA-203a regulates fast muscle differentiation by targeting dmrt2a in zebrafish embryos. Gene.

[28] Cai W (2020). Downregulation of lung miR-203a-3p expression by high-altitude hypoxia enhances VEGF/Notch signaling. Aging.

[29] Hsieh CH (2010). MicroRNA profiling in ischemic injury of the gracilis muscle in rats. BMC Musculoskelet. Disord..

[30] Alexander MS (2013). MicroRNA-199a is induced in dystrophic muscle and affects WNT signaling, cell proliferation, and myogenic differentiation. Cell Death Differ..

[31] Waller R (2017). Serum miRNAs miR-206, 143–3p and 374b–5p as potential biomarkers for amyotrophic lateral sclerosis (ALS). Neurobiol. Aging.

[32] Greco S (2012). Deregulated microRNAs in myotonic dystrophy type 2. PLoS One.

[33] de Mendonca M (2020). MicroRNA miR-222 mediates pioglitazone beneficial effects on skeletal muscle of diet-induced obese mice. Mol. Cell. Endocrinol..

[34] Ritchie W (2017). microRNA target prediction. Methods Mol. Biol..

[35] Guiraud S (2019). The potential of utrophin and dystrophin combination therapies for Duchenne muscular dystrophy. Hum. Mol. Genet..

[36] Pisani C (2018). Utrophin up-regulation by artificial transcription factors induces muscle rescue and impacts the neuromuscular junction in mdx mice. Biochim. Biophys. Acta Mol. Basis Dis..

[37] Polge C (2016). UBE2D2 is not involved in MuRF1-dependent muscle wasting during hindlimb suspension. Int. J. Biochem. Cell. Biol..

[38] Wang F, Zhao B (2019). UBA6 and its bispecific pathways for ubiquitin and FAT10. Int. J. Mol. Sci..

[39] Cardote TAF, Gadd MS, Ciulli A (2017). Crystal structure of the Cul2-Rbx1-EloBC-VHL ubiquitin ligase complex. Structure.

[40] Majmundar AJ (2015). HIF modulation of Wnt signaling regulates skeletal myogenesis in vivo. Development.

[41] Abrigo J (2018). TGF-beta requires the activation of canonical and non-canonical signalling pathways to induce skeletal muscle atrophy. Biol. Chem..

[42] Gonzalez D (2017). ALS skeletal muscle shows enhanced TGF-beta signaling, fibrosis and induction of fibro/adipogenic progenitor markers. PLoS One.

[43] MacDonald EM, Cohn RD (2012). TGFbeta signaling: Its role in fibrosis formation and myopathies. Curr. Opin. Rheumatol..

[44] Turner DC, Seaborne RA, Sharples AP (2019). Comparative transcriptome and methylome analysis in human skeletal muscle anabolism, hypertrophy epigenetic memory. Sci. Rep..

[45] Nikooie R, Jafari-Sardoie S, Sheibani V, Nejadvaziri Chatroudi A (2020). Resistance training-induced muscle hypertrophy is mediated by TGF-beta1-Smad signaling pathway in male Wistar rats. J. Cell. Physiol..

[46] Kanehisa M, Goto S (2000). KEGG: kyoto encyclopedia of genes and genomes. Nucleic Acids Res..

[47] Kanehisa M (2019). Toward understanding the origin and evolution of cellular organisms. Protein Sci..

[48] Kanehisa M, Furumichi M, Sato Y, Kawashima M, Ishiguro-Watanabe M (2023). KEGG for taxonomy-based analysis of pathways and genomes. Nucleic Acids Res..

